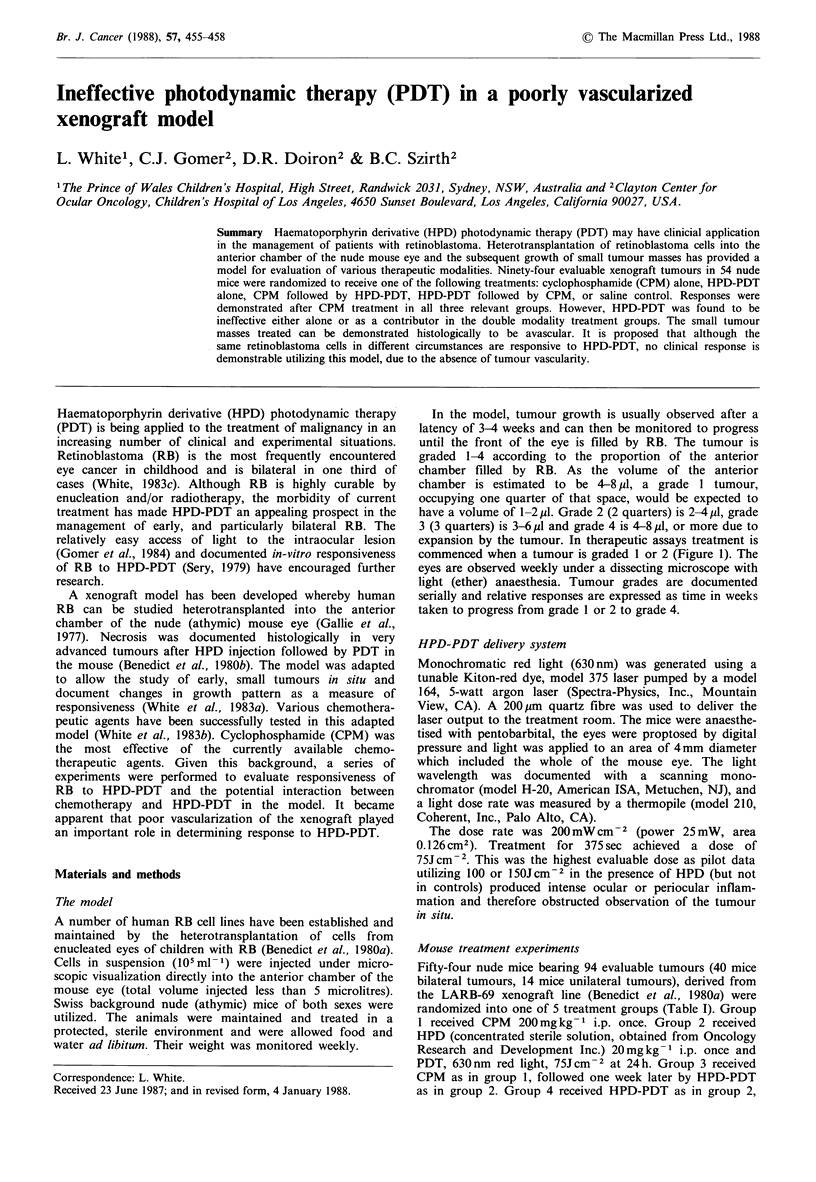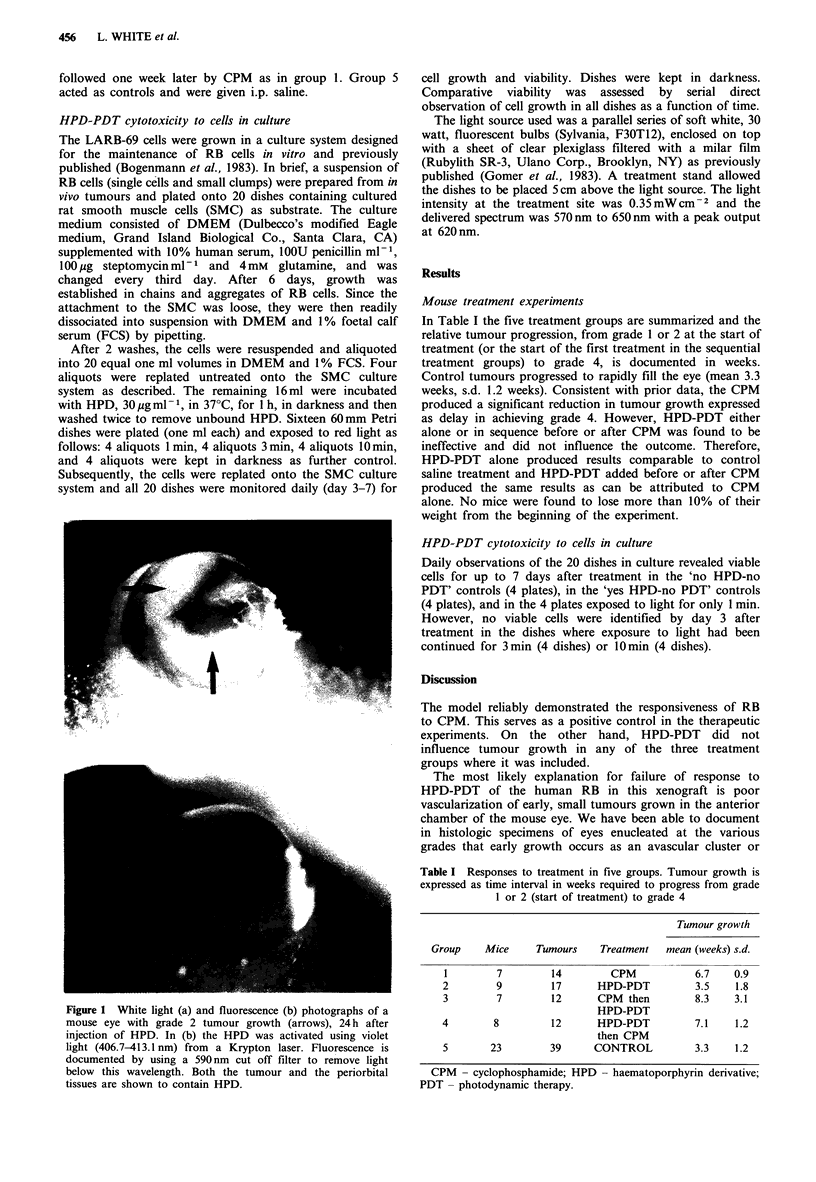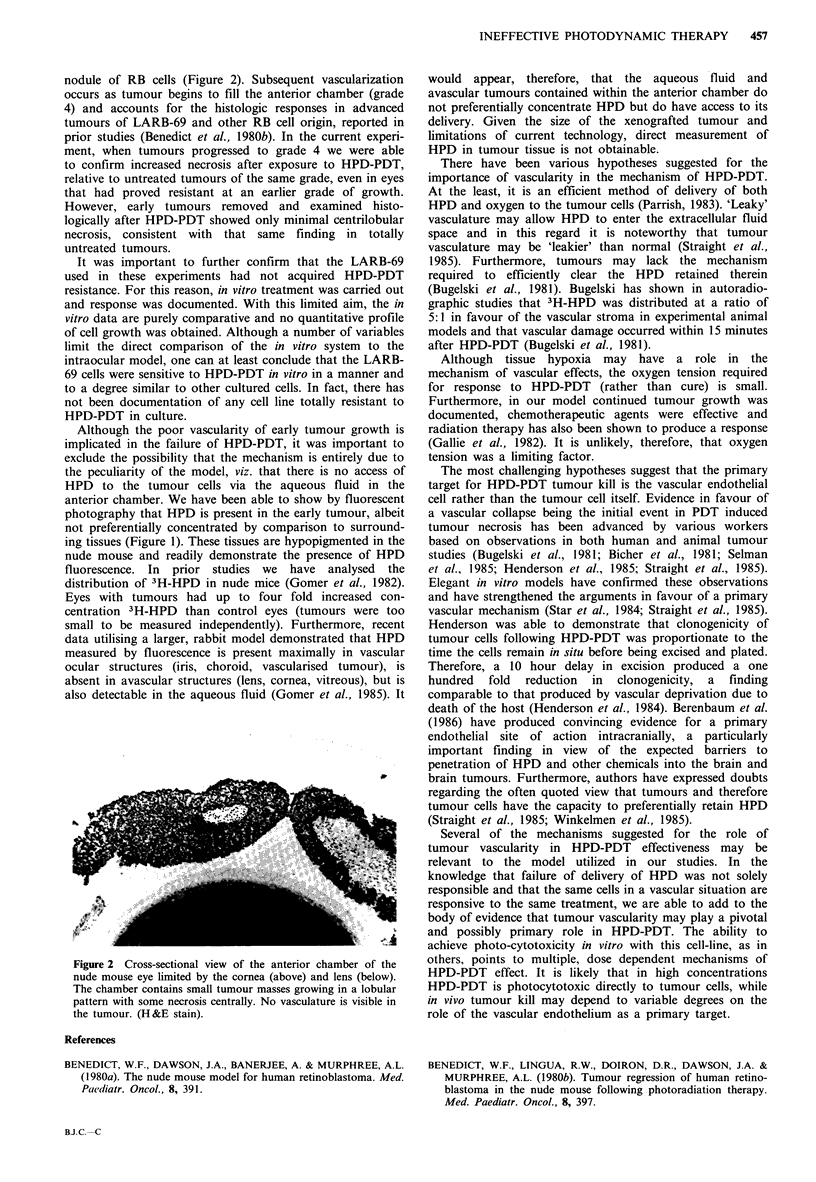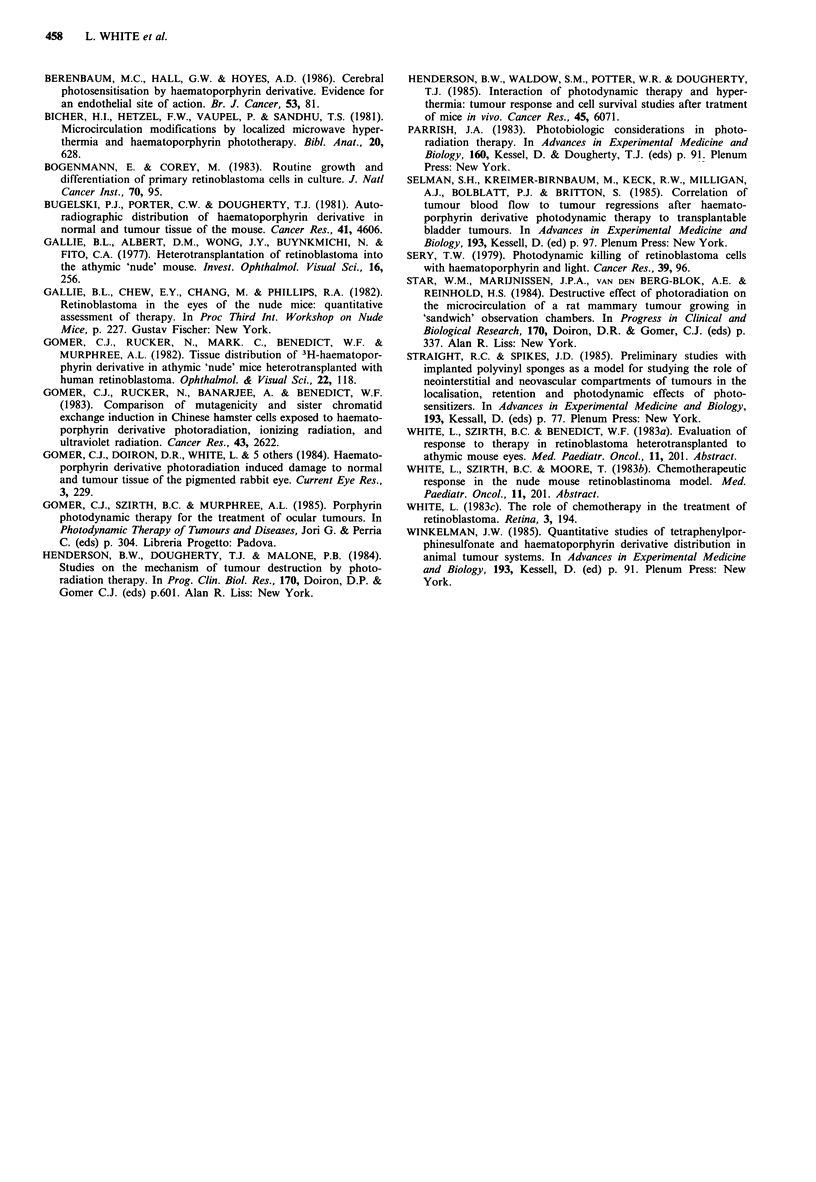# Ineffective photodynamic therapy (PDT) in a poorly vascularized xenograft model.

**DOI:** 10.1038/bjc.1988.106

**Published:** 1988-05

**Authors:** L. White, C. J. Gomer, D. R. Doiron, B. C. Szirth

**Affiliations:** Prince of Wales Children's Hospital, Randwick, Sydney, NSW, Australia.

## Abstract

**Images:**


Br. J. Cancer (1988), 57, 455–458 © The Macmillan Press Ltd., 1988

### Ineffective photodynamic therapy (PDT) in a poorly vascularized xenograft model

L. White[1], C.J. Gomer[2], D.R. Doiron[2] & B.C. Szirth[2]

[1]*The Prince of Wales Children's Hospital, High Street, Randwick 2031, Sydney, NSW, Australia and* [2]*Clayton Center for Ocular Oncology, Children's Hospital of Los Angeles, 4650 Sunset Boulevard, Los Angeles, California 90027, USA.*

**Summary** Haematoporphyrin derivative (HPD) photodynamic therapy (PDT) may have clinicial application in the management of patients with retinoblastoma. Heterotransplantation of retinoblastoma cells into the anterior chamber of the nude mouse eye and the subsequent growth of small tumour masses has provided a model for evaluation of various therapeutic modalities. Ninety-four evaluable xenograft tumours in 54 nude mice were randomized to receive one of the following treatments: cyclophosphamide (CPM) alone, HPD-PDT alone, CPM followed by HPD-PDT, HPD-PDT followed by CPM, or saline control. Responses were demonstrated after CPM treatment in all three relevant groups. However, HPD-PDT was found to be ineffective either alone or as a contributor in the double modality treatment groups. The small tumour masses treated can be demonstrated histologically to be avascular. It is proposed that although the same retinoblastoma cells in different circumstances are responsive to HPD-PDT, no clinical response is demonstrable utilizing this model, due to the absence of tumour vascularity.

Haematoporphyrin derivative (HPD) photodynamic therapy (PDT) is being applied to the treatment of malignancy in an increasing number of clinical and experimental situations. Retinoblastoma (RB) is the most frequently encountered eye cancer in childhood and is bilateral in one third of cases (White, 1983*c*). Although RB is highly curable by enucleation and/or radiotherapy, the morbidity of current treatment has made HPD-PDT an appealing prospect in the management of early, and particularly bilateral RB. The relatively easy access of light to the intraocular lesion (Gomer *et al.*, 1984) and documented *in-vitro* responsiveness of RB to HPD-PDT (Sery, 1979) have encouraged further research.

A xenograft model has been developed whereby human RB can be studied heterotransplanted into the anterior chamber of the nude (athymic) mouse eye (Gallie *et al.*, 1977). Necrosis was documented histologically in very advanced tumours after HPD injection followed by PDT in the mouse (Benedict *et al.*, 1980*b*). The model was adapted to allow the study of early, small tumours *in situ* and document changes in growth pattern as a measure of responsiveness (White *et al.*, 1983*a*). Various chemotherapeutic agents have been successfully tested in this adapted model (White *et al.*, 1983*b*). Cyclophosphamide (CPM) was the most effective of the currently available chemotherapeutic agents. Given this background, a series of experiments were performed to evaluate responsiveness of RB to HPD-PDT and the potential interaction between chemotherapy and HPD-PDT in the model. It became apparent that poor vascularization of the xenograft played an important role in determining response to HPD-PDT.

### Materials and methods

### *The model*

A number of human RB cell lines have been established and maintained by the heterotransplantation of cells from enucleated eyes of children with RB (Benedict *et al.*, 1980*a*). Cells in suspension ($10^5\,ml^{-1}$) were injected under microscopic visualization directly into the anterior chamber of the mouse eye (total volume injected less than 5 microlitres). Swiss background nude (athymic) mice of both sexes were utilized. The animals were maintained and treated in a protected, sterile environment and were allowed food and water *ad libitum*. Their weight was monitored weekly.

Correspondence: L. White.
Received 23 June 1987; and in revised form, 4 January 1988.

In the model, tumour growth is usually observed after a latency of 3–4 weeks and can then be monitored to progress until the front of the eye is filled by RB. The tumour is graded 1–4 according to the proportion of the anterior chamber filled by RB. As the volume of the anterior chamber is estimated to be 4–8 $\mu$l, a grade 1 tumour, occupying one quarter of that space, would be expected to have a volume of 1–2 $\mu$l. Grade 2 (2 quarters) is 2–4 $\mu$l, grade 3 (3 quarters) is 3–6 $\mu$l and grade 4 is 4–8 $\mu$l, or more due to expansion by the tumour. In therapeutic assays treatment is commenced when a tumour is graded 1 or 2 (Figure 1). The eyes are observed weekly under a dissecting microscope with light (ether) anaesthesia. Tumour grades are documented serially and relative responses are expressed as time in weeks taken to progress from grade 1 or 2 to grade 4.

### *HPD-PDT delivery system*

Monochromatic red light (630 nm) was generated using a tunable Kiton-red dye, model 375 laser pumped by a model 164, 5-watt argon laser (Spectra-Physics, Inc., Mountain View, CA). A 200 $\mu$m quartz fibre was used to deliver the laser output to the treatment room. The mice were anaesthetised with pentobarbital, the eyes were proptosed by digital pressure and light was applied to an area of 4 mm diameter which included the whole of the mouse eye. The light wavelength was documented with a scanning monochromator (model H-20, American ISA, Metuchen, NJ), and a light dose rate was measured by a thermopile (model 210, Coherent, Inc., Palo Alto, CA).

The dose rate was $200\,mW\,cm^{-2}$ (power 25 mW, area $0.126\,cm^2$). Treatment for 375 sec achieved a dose of $75\,J\,cm^{-2}$. This was the highest evaluable dose as pilot data utilizing 100 or $150\,J\,cm^{-2}$ in the presence of HPD (but not in controls) produced intense ocular or periocular inflammation and therefore obstructed observation of the tumour *in situ*.

### *Mouse treatment experiments*

Fifty-four nude mice bearing 94 evaluable tumours (40 mice bilateral tumours, 14 mice unilateral tumours), derived from the LARB-69 xenograft line (Benedict *et al.*, 1980*a*) were randomized into one of 5 treatment groups (Table I). Group 1 received CPM $200\,mg\,kg^{-1}$ i.p. once. Group 2 received HPD (concentrated sterile solution, obtained from Oncology Research and Development Inc.) $20\,mg\,kg^{-1}$ i.p. once and PDT, 630 nm red light, $75\,J\,cm^{-2}$ at 24 h. Group 3 received CPM as in group 1, followed one week later by HPD-PDT as in group 2. Group 4 received HPD-PDT as in group 2, followed one week later by CPM as in group 1. Group 5 acted as controls and were given i.p. saline.

*HPD-PDT cytotoxicity to cells in culture*

The LARB-69 cells were grown in a culture system designed for the maintenance of RB cells *in vitro* and previously published (Bogenmann *et al.*, 1983). In brief, a suspension of RB cells (single cells and small clumps) were prepared from *in vivo* tumours and plated onto 20 dishes containing cultured rat smooth muscle cells (SMC) as substrate. The culture medium consisted of DMEM (Dulbecco's modified Eagle medium, Grand Island Biological Co., Santa Clara, CA) supplemented with 10% human serum, 100U penicillin $ml^{-1}$, 100 $\mu g$ steptomycin $ml^{-1}$ and 4 mM glutamine, and was changed every third day. After 6 days, growth was established in chains and aggregates of RB cells. Since the attachment to the SMC was loose, they were then readily dissociated into suspension with DMEM and 1% foetal calf serum (FCS) by pipetting.

After 2 washes, the cells were resuspended and aliquoted into 20 equal one ml volumes in DMEM and 1% FCS. Four aliquots were replated untreated onto the SMC culture system as described. The remaining 16 ml were incubated with HPD, 30 $\mu g\,ml^{-1}$, in 37°C, for 1 h, in darkness and then washed twice to remove unbound HPD. Sixteen 60 mm Petri dishes were plated (one ml each) and exposed to red light as follows: 4 aliquots 1 min, 4 aliquots 3 min, 4 aliquots 10 min, and 4 aliquots were kept in darkness as further control. Subsequently, the cells were replated onto the SMC culture system and all 20 dishes were monitored daily (day 3–7) for cell growth and viability. Dishes were kept in darkness. Comparative viability was assessed by serial direct observation of cell growth in all dishes as a function of time.

The light source used was a parallel series of soft white, 30 watt, fluorescent bulbs (Sylvania, F30T12), enclosed on top with a sheet of clear plexiglass filtered with a milar film (Rubylith SR-3, Ulano Corp., Brooklyn, NY) as previously published (Gomer *et al.*, 1983). A treatment stand allowed the dishes to be placed 5 cm above the light source. The light intensity at the treatment site was 0.35 mW $cm^{-2}$ and the delivered spectrum was 570 nm to 650 nm with a peak output at 620 nm.

**Figure 1** White light (a) and fluorescence (b) photographs of a mouse eye with grade 2 tumour growth (arrows), 24 h after injection of HPD. In (b) the HPD was activated using violet light (406.7–413.1 nm) from a Krypton laser. Fluorescence is documented by using a 590 nm cut off filter to remove light below this wavelength. Both the tumour and the periorbital tissues are shown to contain HPD.

### Results

*Mouse treatment experiments*

In Table I the five treatment groups are summarized and the relative tumour progression, from grade 1 or 2 at the start of treatment (or the start of the first treatment in the sequential treatment groups) to grade 4, is documented in weeks. Control tumours progressed to rapidly fill the eye (mean 3.3 weeks, s.d. 1.2 weeks). Consistent with prior data, the CPM produced a significant reduction in tumour growth expressed as delay in achieving grade 4. However, HPD-PDT either alone or in sequence before or after CPM was found to be ineffective and did not influence the outcome. Therefore, HPD-PDT alone produced results comparable to control saline treatment and HPD-PDT added before or after CPM produced the same results as can be attributed to CPM alone. No mice were found to lose more than 10% of their weight from the beginning of the experiment.

*HPD-PDT cytotoxicity to cells in culture*

Daily observations of the 20 dishes in culture revealed viable cells for up to 7 days after treatment in the 'no HPD-no PDT' controls (4 plates), in the 'yes HPD-no PDT' controls (4 plates), and in the 4 plates exposed to light for only 1 min. However, no viable cells were identified by day 3 after treatment in the dishes where exposure to light had been continued for 3 min (4 dishes) or 10 min (4 dishes).

### Discussion

The model reliably demonstrated the responsiveness of RB to CPM. This serves as a positive control in the therapeutic experiments. On the other hand, HPD-PDT did not influence tumour growth in any of the three treatment groups where it was included.

The most likely explanation for failure of response to HPD-PDT of the human RB in this xenograft is poor vascularization of early, small tumours grown in the anterior chamber of the mouse eye. We have been able to document in histologic specimens of eyes enucleated at the various grades that early growth occurs as an avascular cluster or

**Table I** Responses to treatment in five groups. Tumour growth is expressed as time interval in weeks required to progress from grade 1 or 2 (start of treatment) to grade 4

| Group | Mice | Tumours | Treatment | Tumour growth mean (weeks) | s.d. |
|---|---|---|---|---|---|
| 1 | 7 | 14 | CPM | 6.7 | 0.9 |
| 2 | 9 | 17 | HPD-PDT | 3.5 | 1.8 |
| 3 | 7 | 12 | CPM then HPD-PDT | 8.3 | 3.1 |
| 4 | 8 | 12 | HPD-PDT then CPM | 7.1 | 1.2 |
| 5 | 23 | 39 | CONTROL | 3.3 | 1.2 |

CPM – cyclophosphamide; HPD – haematoporphyrin derivative; PDT – photodynamic therapy.

nodule of RB cells (Figure 2). Subsequent vascularization occurs as tumour begins to fill the anterior chamber (grade 4) and accounts for the histologic responses in advanced tumours of LARB-69 and other RB cell origin, reported in prior studies (Benedict *et al.*, 1980*b*). In the current experiment, when tumours progressed to grade 4 we were able to confirm increased necrosis after exposure to HPD-PDT, relative to untreated tumours of the same grade, even in eyes that had proved resistant at an earlier grade of growth. However, early tumours removed and examined histologically after HPD-PDT showed only minimal centrilobular necrosis, consistent with that same finding in totally untreated tumours.

It was important to further confirm that the LARB-69 used in these experiments had not acquired HPD-PDT resistance. For this reason, *in vitro* treatment was carried out and response was documented. With this limited aim, the *in vitro* data are purely comparative and no quantitative profile of cell growth was obtained. Although a number of variables limit the direct comparison of the *in vitro* system to the intraocular model, one can at least conclude that the LARB-69 cells were sensitive to HPD-PDT *in vitro* in a manner and to a degree similar to other cultured cells. In fact, there has not been documentation of any cell line totally resistant to HPD-PDT in culture.

Although the poor vascularity of early tumour growth is implicated in the failure of HPD-PDT, it was important to exclude the possibility that the mechanism is entirely due to the peculiarity of the model, *viz.* that there is no access of HPD to the tumour cells via the aqueous fluid in the anterior chamber. We have been able to show by fluorescent photography that HPD is present in the early tumour, albeit not preferentially concentrated by comparison to surrounding tissues (Figure 1). These tissues are hypopigmented in the nude mouse and readily demonstrate the presence of HPD fluorescence. In prior studies we have analysed the distribution of $^{3}$H-HPD in nude mice (Gomer *et al.*, 1982). Eyes with tumours had up to four fold increased concentration $^{3}$H-HPD than control eyes (tumours were too small to be measured independently). Furthermore, recent data utilising a larger, rabbit model demonstrated that HPD measured by fluorescence is present maximally in vascular ocular structures (iris, choroid, vascularised tumour), is absent in avascular structures (lens, cornea, vitreous), but is also detectable in the aqueous fluid (Gomer *et al.*, 1985). It would appear, therefore, that the aqueous fluid and avascular tumours contained within the anterior chamber do not preferentially concentrate HPD but do have access to its delivery. Given the size of the xenografted tumour and limitations of current technology, direct measurement of HPD in tumour tissue is not obtainable.

**Figure 2** Cross-sectional view of the anterior chamber of the nude mouse eye limited by the cornea (above) and lens (below). The chamber contains small tumour masses growing in a lobular pattern with some necrosis centrally. No vasculature is visible in the tumour. (H&E stain).

There have been various hypotheses suggested for the importance of vascularity in the mechanism of HPD-PDT. At the least, it is an efficient method of delivery of both HPD and oxygen to the tumour cells (Parrish, 1983). 'Leaky' vasculature may allow HPD to enter the extracellular fluid space and in this regard it is noteworthy that tumour vasculature may be 'leakier' than normal (Straight *et al.*, 1985). Furthermore, tumours may lack the mechanism required to efficiently clear the HPD retained therein (Bugelski *et al.*, 1981). Bugelski has shown in autoradiographic studies that $^{3}$H-HPD was distributed at a ratio of 5:1 in favour of the vascular stroma in experimental animal models and that vascular damage occurred within 15 minutes after HPD-PDT (Bugelski *et al.*, 1981).

Although tissue hypoxia may have a role in the mechanism of vascular effects, the oxygen tension required for response to HPD-PDT (rather than cure) is small. Furthermore, in our model continued tumour growth was documented, chemotherapeutic agents were effective and radiation therapy has also been shown to produce a response (Gallie *et al.*, 1982). It is unlikely, therefore, that oxygen tension was a limiting factor.

The most challenging hypotheses suggest that the primary target for HPD-PDT tumour kill is the vascular endothelial cell rather than the tumour cell itself. Evidence in favour of a vascular collapse being the initial event in PDT induced tumour necrosis has been advanced by various workers based on observations in both human and animal tumour studies (Bugelski *et al.*, 1981; Bicher *et al.*, 1981; Selman *et al.*, 1985; Henderson *et al.*, 1985; Straight *et al.*, 1985). Elegant *in vitro* models have confirmed these observations and have strengthened the arguments in favour of a primary vascular mechanism (Star *et al.*, 1984; Straight *et al.*, 1985). Henderson was able to demonstrate that clonogenicity of tumour cells following HPD-PDT was proportionate to the time the cells remain *in situ* before being excised and plated. Therefore, a 10 hour delay in excision produced a one hundred fold reduction in clonogenicity, a finding comparable to that produced by vascular deprivation due to death of the host (Henderson *et al.*, 1984). Berenbaum *et al.* (1986) have produced convincing evidence for a primary endothelial site of action intracranially, a particularly important finding in view of the expected barriers to penetration of HPD and other chemicals into the brain and brain tumours. Furthermore, authors have expressed doubts regarding the often quoted view that tumours and therefore tumour cells have the capacity to preferentially retain HPD (Straight *et al.*, 1985; Winkelmen *et al.*, 1985).

Several of the mechanisms suggested for the role of tumour vascularity in HPD-PDT effectiveness may be relevant to the model utilized in our studies. In the knowledge that failure of delivery of HPD was not solely responsible and that the same cells in a vascular situation are responsive to the same treatment, we are able to add to the body of evidence that tumour vascularity may play a pivotal and possibly primary role in HPD-PDT. The ability to achieve photo-cytotoxicity *in vitro* with this cell-line, as in others, points to multiple, dose dependent mechanisms of HPD-PDT effect. It is likely that in high concentrations HPD-PDT is photocytotoxic directly to tumour cells, while *in vivo* tumour kill may depend to variable degrees on the role of the vascular endothelium as a primary target.

### References

BENEDICT, W.F., DAWSON, J.A., BANERJEE, A. & MURPHREE, A.L. (1980*a*). The nude mouse model for human retinoblastoma. *Med. Paediatr. Oncol.*, **8**, 391.

BENEDICT, W.F., LINGUA, R.W., DOIRON, D.R., DAWSON, J.A. & MURPHREE, A.L. (1980*b*). Tumour regression of human retinoblastoma in the nude mouse following photoradiation therapy. *Med. Paediatr. Oncol.*, **8**, 397.

BERENBAUM, M.C., HALL, G.W. & HOYES, A.D. (1986). Cerebral photosensitisation by haematoporphyrin derivative. Evidence for an endothelial site of action. *Br. J. Cancer*, **53**, 81.

BICHER, H.I., HETZEL, F.W., VAUPEL, P. & SANDHU, T.S. (1981). Microcirculation modifications by localized microwave hyperthermia and haematoporphyrin phototherapy. *Bibl. Anat.*, **20**, 628.

BOGENMANN, E. & COREY, M. (1983). Routine growth and differentiation of primary retinoblastoma cells in culture. *J. Natl Cancer Inst.*, **70**, 95.

BUGELSKI, P.J., PORTER, C.W. & DOUGHERTY, T.J. (1981). Autoradiographic distribution of haematoporphyrin derivative in normal and tumour tissue of the mouse. *Cancer Res.*, **41**, 4606.

GALLIE, B.L., ALBERT, D.M., WONG, J.Y., BUYNKMICHI, N. & FITO, C.A. (1977). Heterotransplantation of retinoblastoma into the athymic 'nude' mouse. *Invest. Ophthalmol. Visual Sci.*, **16**, 256.

GALLIE, B.L., CHEW, E.Y., CHANG, M. & PHILLIPS, R.A. (1982). Retinoblastoma in the eyes of the nude mice: quantitative assessment of therapy. In *Proc Third Int. Workshop on Nude Mice*, p. 227. Gustav Fischer: New York.

GOMER, C.J., RUCKER, N., MARK. C., BENEDICT, W.F. & MURPHREE, A.L. (1982). Tissue distribution of $^3$H-haematoporphyrin derivative in athymic 'nude' mice heterotransplanted with human retinoblastoma. *Ophthalmol. & Visual Sci.*, **22**, 118.

GOMER, C.J., RUCKER, N., BANARJEE, A. & BENEDICT, W.F. (1983). Comparison of mutagenicity and sister chromatid exchange induction in Chinese hamster cells exposed to haematoporphyrin derivative photoradiation, ionizing radiation, and ultraviolet radiation. *Cancer Res.*, **43**, 2622.

GOMER, C.J., DOIRON, D.R., WHITE, L. & 5 others (1984). Haematoporphyrin derivative photoradiation induced damage to normal and tumour tissue of the pigmented rabbit eye. *Current Eye Res.*, **3**, 229.

GOMER, C.J., SZIRTH, B.C. & MURPHREE, A.L. (1985). Porphyrin photodynamic therapy for the treatment of ocular tumours. In *Photodynamic Therapy of Tumours and Diseases*, Jori G. & Perria C. (eds) p. 304. Libreria Progetto: Padova.

HENDERSON, B.W., DOUGHERTY, T.J. & MALONE, P.B. (1984). Studies on the mechanism of tumour destruction by photoradiation therapy. In *Prog. Clin. Biol. Res.*, **170**, Doiron, D.P. & Gomer C.J. (eds) p.601. Alan R. Liss: New York.

HENDERSON, B.W., WALDOW, S.M., POTTER, W.R. & DOUGHERTY, T.J. (1985). Interaction of photodynamic therapy and hyperthermia: tumour response and cell survival studies after tratment of mice *in vivo*. *Cancer Res.*, **45**, 6071.

PARRISH, J.A. (1983). Photobiologic considerations in photoradiation therapy. In *Advances in Experimental Medicine and Biology*, **160**, Kessel, D. & Dougherty, T.J. (eds) p. 91. Plenum Press: New York.

SELMAN, S.H., KREIMER-BIRNBAUM, M., KECK, R.W., MILLIGAN, A.J., BOLBLATT, P.J. & BRITTON, S. (1985). Correlation of tumour blood flow to tumour regressions after haematoporphyrin derivative photodynamic therapy to transplantable bladder tumours. In *Advances in Experimental Medicine and Biology*, **193**, Kessell, D. (ed) p. 97. Plenum Press: New York.

SERY, T.W. (1979). Photodynamic killing of retinoblastoma cells with haematoporphyrin and light. *Cancer Res.*, **39**, 96.

STAR, W.M., MARIJNISSEN, J.P.A., VAN DEN BERG-BLOK, A.E. & REINHOLD, H.S. (1984). Destructive effect of photoradiation on the microcirculation of a rat mammary tumour growing in 'sandwich' observation chambers. In *Progress in Clinical and Biological Research*, **170**, Doiron, D.R. & Gomer, C.J. (eds) p. 337. Alan R. Liss: New York.

STRAIGHT, R.C. & SPIKES, J.D. (1985). Preliminary studies with implanted polyvinyl sponges as a model for studying the role of neointerstitial and neovascular compartments of tumours in the localisation, retention and photodynamic effects of photosensitizers. In *Advances in Experimental Medicine and Biology*, **193**, Kessall, D. (eds) p. 77. Plenum Press: New York.

WHITE, L., SZIRTH, B.C. & BENEDICT, W.F. (1983*a*). Evaluation of response to therapy in retinoblastoma heterotransplanted to athymic mouse eyes. *Med. Paediatr. Oncol.*, **11**, 201. *Abstract.*

WHITE, L., SZIRTH, B.C. & MOORE, T. (1983*b*). Chemotherapeutic response in the nude mouse retinoblastinoma model. *Med. Paediatr. Oncol.*, **11**, 201. *Abstract.*

WHITE, L. (1983*c*). The role of chemotherapy in the treatment of retinoblastoma. *Retina*, **3**, 194.

WINKELMAN, J.W. (1985). Quantitative studies of tetraphenylporphinesulfonate and haematoporphyrin derivative distribution in animal tumour systems. In *Advances in Experimental Medicine and Biology*, **193**, Kessell, D. (ed) p. 91. Plenum Press: New York.